# Improving Eco-Friendly Polymer Adhesive Joints: Innovative Toughening Strategies for Consistent Performance Under Various Loading Conditions

**DOI:** 10.3390/polym17050648

**Published:** 2025-02-28

**Authors:** Shahin Jalali, Ricardo J. C. Carbas, Eduardo A. S. Marques, Lucas F. M. da Silva

**Affiliations:** 1 Institute of Science and Innovation in Mechanical and Industrial Engineering (INEGI), Rua Dr. Roberto Frias, 4200-465 Porto, Portugal; sh.jalali.94@gmail.com; 2 Departamento de Engenharia Mecânica, Faculdade de Engenharia, Universidade do Porto, Rua Dr. Roberto Frias, 4200-465 Porto, Portugal; emarques@fe.up.pt

**Keywords:** bio-joints, wooden joints, toughened joint, transverse strength, delamination

## Abstract

In modern engineering applications, the use of sustainable materials and eco-friendly methods has become increasingly important. Wood joints, especially those strengthened with bio-adhesive, have attracted considerable attention due to their inherent environmental benefits and desirable mechanical properties. Compared to traditional joining methods, adhesive joints offer unique advantages such as improved load distribution, reduced stress concentration, and enhanced aesthetic appeal. This study aims to enhance delamination resistance in wooden adhesive joints using a novel method involving reinforced high-toughness resin on surfaces. Additionally, a hybrid substrate approach applies a tough layer to outer plies and a densified wood core with greater fiber direction strength. Normal, toughened, and hybrid single-lap joint specimens were analyzed through both experimental and numerical methods under various loading conditions, including quasi-static and intermediate rates. The proposed method involved bio-adhesive penetration into the wood substrate, forming a reinforced surface zone. The experimentally validated results show a significant improvement in joint strength, exhibiting an approximate 2.8-fold increase for the toughened joints compared to the reference joints under intermediate-rate conditions. Furthermore, the absorbed energy of the toughened joints increased by a substantial factor of up to 4.5 times under the same conditions. The fracture surfaces analysis revealed that the toughening method changed the failure mechanism of the joints from delamination to fiber breakage, indicating that the strength of the substrate was lower than that of the joint under impact conditions. The viscoelastic behavior of the bio-adhesive also influenced the response of the joints to the changing displacement rate. The toughening method enhanced the resilience and load-bearing capacity of the wood joints, making them more suitable for dynamic applications.

## 1. Introduction

Bio-based materials have gained prominence as sustainable alternatives to fossil-based products, aiming to mitigate the environmental impact of human activities [1,2,3]. Sourced from trees and plants, these materials support the development of eco-friendly structures capable of bearing loads. Notably, natural fiber composites—such as flax, jute, and palm tree fibers—exhibit significant potential for diverse applications. Within this realm, wood, a renewable natural resource that has been utilized for centuries across various domains, warrants particular consideration [4].

Wood has many noteworthy attributes that make it a highly attractive material choice, such as its versatility, durability, resistance, robustness, low weight, abundance, and economic viability. Furthermore, wood can adapt to changing forms under different environmental conditions, such as temperature, loading, and moisture, resulting in intricate artifacts with refined geometries [5,6]. Wood’s ability to transform in response to its surroundings adds value to its utility and enables innovative applications that exploit its unique properties. In this era of sustainability-driven innovation, wood and other plant-based materials play a vital role in creating a greener and more resource-conscious future. Wood is a natural composite material that, like most composites, is sensitive to holes and notches. Therefore, traditional joining methods such as riveting and bolting are unsuitable for wood [7,8]. Adhesive bonding is an effective method for joining wood components, as it minimizes stress concentrations while providing a larger, more evenly distributed bonded area. Although adhesive joints are primarily designed to withstand shear forces, when two substrates are overlapped and subjected to opposing forces, these forces align, generating a flexural moment at the overlap ends. This results in localized stress concentrations and alters the adhesive’s stress distribution from pure shear to a combination of shear and tensile forces. This phenomenon highlights the complex interactions that adhesive joints experience under different loading conditions [9,10]. The cohesive properties of the adhesive and the adhering substrates determine the failure load and mode of the joint. Peel loading is especially problematic for adhesive joints with wood substrates, as it causes delamination between different grain layers and leads to joint failure. To address this problem, researchers have proposed various methods, such as densification, substrate toughening, and physical modifications of the adhesive. These methods aim to prevent delamination and enhance the strength of adhesive joints with wood substrates. The use of adhesives for bonding wood substrates requires a careful approach to ensure the design of effective, safe, and high-performance modern wooden structures. With the increasing application of wooden structures in various fields, such as vehicular engineering, several physical and chemical modifications have been developed to enhance the mechanical properties of these materials. Consequently, adhesively bonded joints have become popular in different industries, such as civil, automotive, and aeronautical engineering, due to their advantages over traditional joints. Recent studies have focused on improving the performance of wooden adhesive joints by using bio-adhesives and wood-based substrates. The main objective of these studies was to characterize these materials and integrate them into adhesive joints. However, a major challenge is the durability of bio-adhesives under harsh environmental conditions, such as high temperature and humidity, over a long service life [11,12].

Addressing this durability challenge assumes significance as it ultimately determines the practicality and longevity of the adhesive joints in real-world scenarios. The failure mode and load capacity of joints are significantly influenced by the properties of both the adhesive and the substrate. Composite materials commonly face a challenge related to the strength of their matrix, particularly when exposed to peel stress. This arises because the fibers within the matrix may not reach their optimal efficiency [13,14]. Therefore, any method that enhances the peeling strength of the wood directly contributes to the overall strength of the joints [10].

The durability of a given bio-adhesive and its additives is still an unexplored path. Tian et al. [15] tried to increase the humidity resistance of soy-protein films by adding polyurethane or natural rubber to the film. Usually, humidity studies involve exposing a thin material plate to a certain humidity condition and measuring its mass changes. However, this method did not work for these films. Instead, the hydrophilicity of the film surface was measured by the contact angle, which showed that adding polyurethane or natural rubber can make the material more hydrophobic and, therefore, more moisture-resistant. Moreover, the thermal stability of soy-based materials was improved by adding inorganic fillers [10,16].

Altuna et al. [17] studied the effect of soybean oil (ESO) on the properties of epoxy resins cured with diglyceryl ether of bisphenol A (DGEBA). The results showed that increasing the ESO content led to a decrease in the storage modulus and glass transition temperature (Tg) of the adhesive. At 100% ESO, the Tg was almost half of that at 0% ESO. This indicates that adding ESO can make the material rubbery at room temperature, which limits its use in applications that require a strong and stiff material.

Baumberger et al. [18] investigated films containing up to 30% kraft lignin and starch which were produced using extrusion and thermal molding. The mechanical properties of the composite were assessed through tensile testing at two different humidity levels. The study revealed that at 58% relative humidity (RH), lignin enhanced both the strength and strain-to-failure of the composite for lignin contents up to 20%. However, at 71% RH, lignin had the opposite effect on the composite’s properties across the 0% to 30% content range. It was also noted that lignin reduced the films’ water affinity, a behavior attributed to the interplay between the hydrophilic starch matrix and the hydrophobic lignin. This characteristic raises concerns in adhesives with additives, as the adhesive matrix is typically more hydrophilic than the reinforcement, making the adhesive/reinforcement interface a potential pathway for water diffusion [19].

One way to improve the peel strength of single-lap joints (SLJ) is by using a matrix with high transverse toughness, which enhances resistance to peel stress. This approach can be complemented by additional techniques designed to minimize local stress concentrations in the composite adherends [20,21]. Ramezani et al. [22] investigated the strength of hybrid composite joints reinforced with various laminate materials. They reviewed several methods to improve SLJ strength, including transverse toughening and high-toughness matrix techniques. The study utilized composite adherends and different laminates, such as aluminum, polymeric, and thin-ply. They found that thin-ply laminates increased joint strength by 10% and reduced peel stress at the overlap ends. The authors suggested that hybrid composite joints with different laminate materials offer benefits in terms of weight savings, damage resistance, and load transfer performance.

Shang et al. [14] proposed a methodology to address delamination issues in adhesive joints with composite substrates. Their investigation included both experimental and numerical evaluations of joint strength, failure modes, and stress distribution within SLJs. The toughened composite material resulted in a significant 22% increase in joint strength and a shift in failure mode from adherend delamination to cohesive failure within the adhesive. The study’s findings indicate that toughened composite materials can effectively mitigate delamination in adhesive joints involving composite substrates while enhancing overall performance.

Bliem et al. [23] investigated the effects of drying temperature, pressing time, and pressing temperature on the shear and tensile strength of wooden single-lap joints. The first step involved keeping the joints at 60 °C and 95 °C for four hours, and the results showed that the 60 °C temperature had no effect on joint strength, while higher temperatures resulted in the degradation of joint strength due to the wood drying. In the second step, the samples were cured at 120 °C and 200 °C for the same duration, and it was found that higher curing temperatures led to a reduction in joint strength due to hemicellulose degradation, while an increase in pressing time caused a change in the failure mode from cohesive to wood delamination. The durability of bio-adhesives and their additives is a poorly understood topic. This study presents a novel technique to improve the mechanical performance of bio-based single-lap joints (SLJs) made of wood substrates and a polyurethane-based bio-adhesive. Wood has some limitations, such as low shear and peel strength, which restrict its use in load-bearing structures. To address this issue, a method to reinforce the wood substrates with bio-adhesive was proposed. This method uses plant-based materials that have high bonding strength and biodegradability. By applying the bio-adhesive on the surface of the wood substrates, the shear and peel strength of the SLJs can increase, as well as the absorbed energy during failure. Experimental tests were performed to evaluate the effectiveness of this technique, comparing it to conventional SLJs made from non-toughened pine wood substrates. A numerical model was also developed to explain the mechanisms behind the mechanical behaviors observed.

## 2. Material and Methods

### 2.1. Bio-Substrate

#### 2.1.1. Pine Wood

Wood, a natural and renewable resource, has been extensively utilized across various industries for centuries due to its abundance and favorable mechanical properties. In this study, pine wood was chosen as the primary substrate, sourced from the southern regions of Portugal. This selection was based on its affordability, wide availability, and appropriate mechanical characteristics. The mechanical behavior of wood is influenced by factors such as species, growth conditions, grain structure, and defects. To ensure consistency, symmetrical, straight-grained beams were used, avoiding knotted timber.

Mechanical and cohesive properties of pine wood, as determined by Oliveira et al. [24], are shown in Table 1 and Table 2. The moisture content ranged from 8% to 25%, with all substrates between 12% and 18% as supplied. Dry wood (≤19% moisture) ensures stability, strength, and biological resistance. Pine timbers, initially 1000 mm long, were cut to 100 mm × 25 mm × 6 mm dimensions for the study.

*E* is defined as Young’s modulus, *ν* is defined as Poisson’s ratio, and *G* is defined as shear modulus.

#### 2.1.2. Densified Pine Wood

Pine wood blocks (45 × 40 mm, average length 240 mm) were densified using a two-step process adapted from Song et al. [23]. In the first step, the blocks were boiled for seven hours in a chemical solution containing 2.5 M NaOH and 0.4 M Na_2_SO_3_. This treatment enabled the chemical catalyst to penetrate the cell walls, increasing their volume by breaking down hemicellulose and lignin. Next, the blocks were boiled in deionized water for 1 h to remove residual chemicals, with the water replaced every 30 min. This step further expanded the cell volume by filling the empty spaces with water.

**Figure 1:**
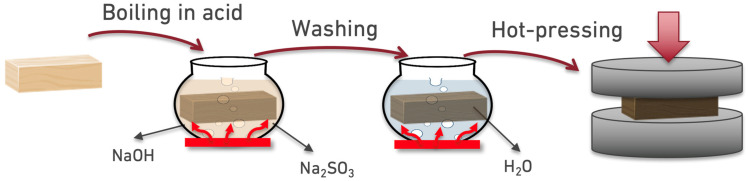
Densification process of wood.

The second step involved thermo-mechanical processing. The blocks were hot-pressed in a steel mold for 24 h at 100 °C under 3 MPa pressure. This compressed the cell walls, causing them to collapse while preserving the fibers, resulting in greater density and strength (see Figure 1).

Maintaining moisture content (12–20%) was essential to preserve the wood’s strength, flexibility, and stability. Moisture control was achieved through conditioning in controlled humidity and temperature environments. Densified wood blocks were further treated in silica gel (2–5 mm diameter) with a moisture indicator, at 70 °C for 48 h. This harmonized the moisture levels, ensuring durability and resistance to chemical reactions while preventing warping or cracking.

### 2.2. Bio-Adhesive

Structural adhesives have traditionally been derived from petroleum-based resources; however, increasing concerns regarding environmental, health, and economic impacts have driven a transition toward more sustainable alternatives. Among these, bio-based adhesives have gained recognition for their advantages, including lower toxicity, a reduced environmental footprint, and potential cost savings compared to fossil-based counterparts.

This study examined a moisture-cured polyurethane bio-adhesive, which is particularly noteworthy for being composed of 70% natural resources. It enhances bond strength through a combination of mechanical interlocking and chemical bonding with wooden substrates, specifically through interactions between hydroxyl (OH) groups in the wood and the adhesive’s components. This dual bonding mechanism significantly improves the adhesive’s overall performance.

However, at high moisture levels (12–20%), the primary curing mechanism shifts toward the reaction of isocyanate (–NCO) groups with water, resulting in the formation of polyurea and biuret networks, rather than –NCO reacting with hydroxyls.

Moisture content of the wood is critical to the adhesive’s curing process, as it directly affects curing time and performance. To ensure consistent results, all wooden substrates were maintained at a moisture content of 12–20%, a range recommended by the supplier. Proper moisture control not only optimized the curing process but also improved the adhesive’s reliability and performance. The bio-adhesive was cured in two stages: first at 100 °C for 8 then for 48 h at room temperature as the post-curing process. The mechanical properties of the bio-adhesive are presented in Table 3.

### 2.3. Joint Manufacturing and Toughening Procedures

**Figure 2:**
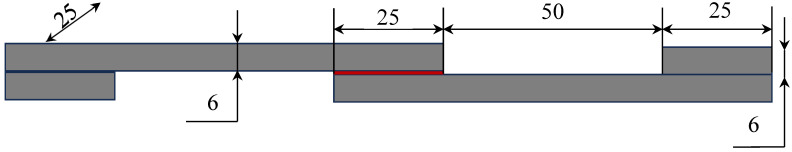
Schematic representation of SLJ geometry and dimensions (dimensions in mm).

To manufacture the SLJ specimens, a 25 mm overlap length was selected, ensuring direct bonding of substrates under pressure to meet the zero-thickness requirement for bio-adhesive application. Clamps were used to ensure uniform pressure across the bonding area, while 2 mm thick aluminum plates were placed on the outer surfaces to protect the substrates from damage. A mold-release agent was applied to the aluminum surfaces for easy detachment. Additionally, wooden end tabs were bonded to the joints using Araldite AV138 manufactured by Huntsman Advanced Materials, Switzerland, a two-component epoxy adhesive, which cured at room temperature in 24 h. Figure 2 illustrates the joint geometries and dimensions.

Assuming minimal edge effects, the stress and strain along the width should remain consistent, meaning the failure load of the SLJs is directly proportional to the specimen width. To ensure uniformity and allow for effective comparisons, a specimen width of 25 mm was chosen for this study. A comprehensive sanding with 400-grade sandpaper created smooth, uniform surfaces for strong bonding. After sanding, dust particles were removed using compressed air to prevent interference with the adhesive. To ensure complete cleanliness, acetone was also used for an additional cleaning step. Once the surfaces were prepared, a layer of adhesive was applied before assembling the substrates. Simultaneously, six SLJs were produced and placed in an oven for curing, allowing the adhesive to bond and solidify. After curing, any excess adhesive was carefully removed to maintain a clean appearance.

**Figure 3:**
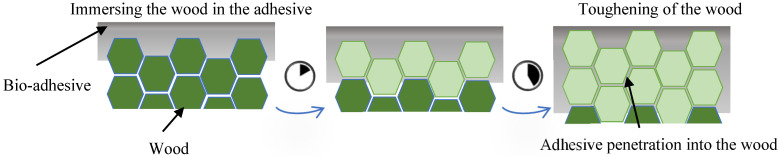
Substrate-toughening procedure.

To enhance the robustness of substrate edges, an adhesive infusion process was applied, leveraging the superior strength of the bio-adhesive compared to the wood matrix, especially lignin. The process involved two phases. In the first phase, the substrate surface was evenly coated with the bio-adhesive using a brush. This ensured uniform distribution and thorough absorption into the wood fibers, creating a solid foundation for bonding. In the second phase, the substrate was immersed in an adhesive bath for an hour. This step allowed deeper penetration of the adhesive into the wood, reaching otherwise inaccessible areas and reinforcing the bond (see Figure 3). This method significantly strengthened the adhesive–wood interaction, as reflected in the mechanical properties shown in Table 4 [26].

**Figure 4:**
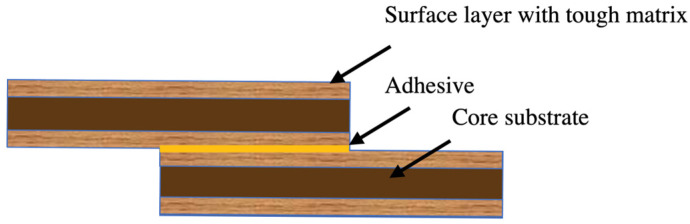
Schematic of surface toughening.

**Figure 5:**
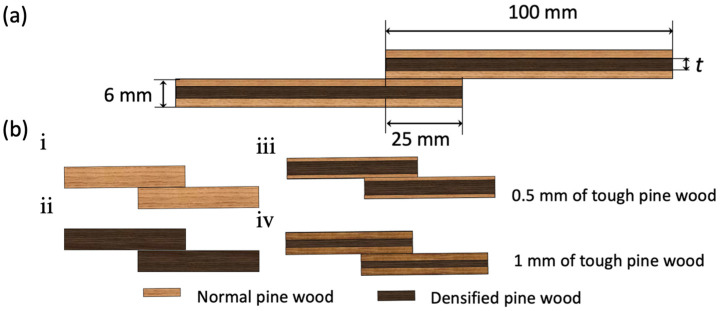
SLJ geometry, where t is the densified wood thickness (0, 4, 5, and 6 mm) (**a**), and tested joint configurations (**b**).

Both composite laminates and wood structures are susceptible to premature failure due to delamination. However, their transverse strength can be increased by employing toughening methods that lead to structures with improved strength, greater fatigue life, and higher resistance to delamination [27]. In high-performance structures, brittle substrates usually result in high stress concentrations. By using plies with stiffer properties along the transverse direction at the outer layers of composite laminates, the peel stress levels in modern composite and adhesive joint design can be effectively reduced [28]. One of the most simple and effective methods for increasing the transverse strength of laminated structures is to use a toughened layer, which reduces the peel stress level. To achieve this, a thin layer of a material with higher toughness is applied to the surfaces of the laminated structures where peel stress is at the highest level, as compared to other areas throughout the thickness of the adherents. This delays composite delamination and improves the transverse strength of the entire laminated structure. In ideal conditions, the failure mode changes from delamination in the laminated structure to cohesive failure in the adhesive, which is even better. Figure 4 shows surface toughening of a single-lap joint specimen (Figure 5a). To compare the effects of tough layer thickness, two different thicknesses (0.5 mm and 1 mm) were used to manufacture the substrates. Various configurations were tested, as shown in Figure 5b:

(i) Reference joint with normal wood,
(ii) Reference joint with densified wood,
(iii) Hybrid joint with 5 mm densified wood core and 0.5 mm normal wood on each side,
(iv) Hybrid joint with 4 mm densified wood core and 1 mm normal wood on each side.

These configurations were analyzed to assess the impact of the tough layer on joint performance, with details shown in Figure 5.

### 2.4. Testing Conditions

Adhesive joints play a vital role in real-world applications, often subjected to static and dynamic loads, including intermediate-rate conditions. These involve forces applied at varying speeds, from gradual to rapid events, such as impacts. Understanding quasi-static and intermediate loading behaviors is essential, as gradual loads can degrade performance over time, while intermediate rates demand resilience for safety in high-stress scenarios like collisions or machinery operation.

Quasi-static tests were conducted using an INSTRON 3367^®^ (manufactured by Instron, Inc., Norwood, MA, USA) universal testing machine with a 30 kN load cell at 1 mm/min. Intermediate rate tests used an Instron 8801 (manufactured by Instron, Inc., Norwood, MA, USA) servo-hydraulic machine with a 100 kN load cell at 6 m/min. Each joint configuration was tested three times at room temperature, generating load–displacement curves to assess mechanical response and consistency.

## 3. Numerical Analysis

**Figure 6:**
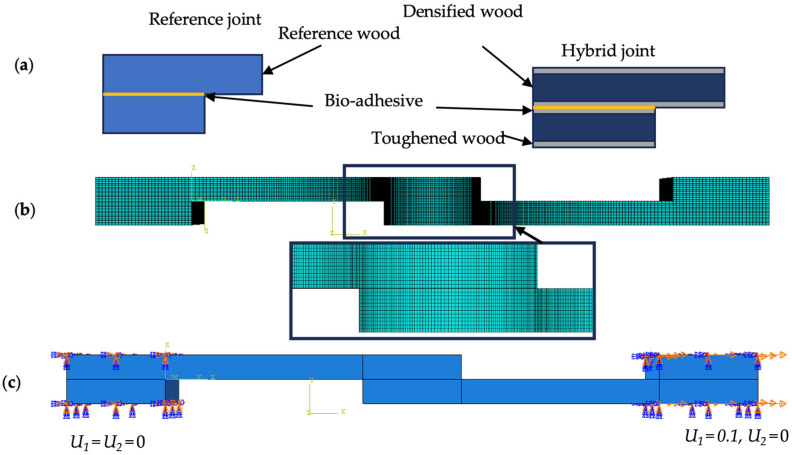
Numerical simulation details. Assigned properties (**a**), mesh used in SLJ simulation (**b**), and boundary condition (**c**).

This section presents the numerical simulation of the SLJ under static and high rate using ABAQUS/Explicit. To streamline the analysis and minimize computational effort, a two-dimensional (2D) model was utilized to replicate the joint’s behavior under static and intermediate loading conditions, with the assumption that stress distribution across the SLJ’s width remains consistent. The objective of the simulation was to simulate the failure mode and the load–displacement curve of the joint, and to compare the stress distribution along the bondline in different configurations. The primary goal was to predict load-displacement behavior and failure modes. Numerical results were compared to a representative experimental curve. Due to strain rate effects on viscoelastic materials, strength improved by 15% under intermediate-rate conditions. Substrates were assembled with a 25 mm overlap using cohesive contact, adhering to the properties in Table 2. The geometry and mesh of the bio–single-lap joint model are shown in Figure 3 and Figure 6. The joint consisted of two wood-based substrate and one bio-adhesive layer. The dimensions of the substrates were 100 mm × 25 mm × 6 mm, and the dimensions of the adhesive layer were 25 mm × 25 mm × 0.02 mm. The material properties of the bio-adhesive and the wood-based substrate were obtained from the experimental tests described in Section 2. The bio-adhesive was assumed to be isotropic and cohesive, with the properties presented in Table 4. The wood-based substrate was assumed to be orthotropic and elastic, with different Young’s moduli, Poisson’s ratios, and shear moduli along the longitudinal, transverse, and normal directions. The values of these parameters are given in Table 1. Cohesive quadrilateral elements with four nodes were used in the model; the smallest elements were 0.3 × 0.01 × 0.5 mm. The mesh was refined near the overlap region to capture the stress concentration and damage evolution. Mesh refinement occurred in regions with significant stress gradients, using single and double biasing (0.01 and 0.05) along the substrates and bondline in the x-direction. Uniform mesh distribution was applied along end tabs (Figure 6c). The boundary conditions were applied by fixing one end of the joint while displacing the other end along the x-direction only. In all tests conducted, the wood samples demonstrated elastic deformation, leading to the presumption of linear elastic behavior for the substrates. The model incorporated quadrilateral mesh elements with four nodes under plane stress conditions. To forecast the onset and growth of potential damage and delamination in the substrates, a cohesive zone model (CZM) was applied. This model utilized four-node cohesive quadrilateral elements to represent the substrate behavior, adopting a bilinear traction-separation law shaped by the mechanical and cohesive characteristics of pine wood. The CZM elements, accounting for the experimentally observed delamination thickness, were integrated into both standard and toughened substrates to accurately depict the delamination process. The cohesive elements’ thickness was determined based on the average delamination thickness recorded in the experiments. For simulating adhesive behavior, cohesive contact was established between the two substrates, reflecting the properties of the bio-adhesive. In areas experiencing pronounced stress gradients, mesh refinement was achieved through size reduction of the elements.

## 4. Results and Discussion

In this section, the peel stress distribution of adhesive single-lap joints with various wood-based substrates is first discussed. Subsequently, the intermediate rate and quasi-static behaviors of the joints are presented. Finally, the fracture surfaces are analyzed to understand the failure mechanisms. The peel stress behavior of these joints was examined under both dynamic and static loading conditions to evaluate the effects of substrate modifications on the performance of the adhesive joints. For the dynamic case, stress distribution was captured at a displacement of 0.5 mm, while for the static case, it was analyzed under a constant applied load. Various configurations of wood-based substrates were considered, and the fracture surfaces were studied to correlate the stress distribution with the observed failure modes.

Peel stress analysis under static loading conditions

**Figure 7:**
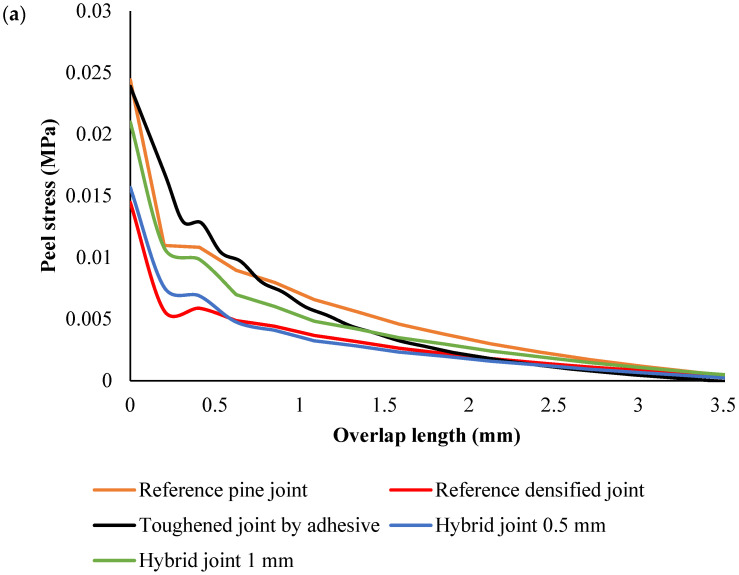

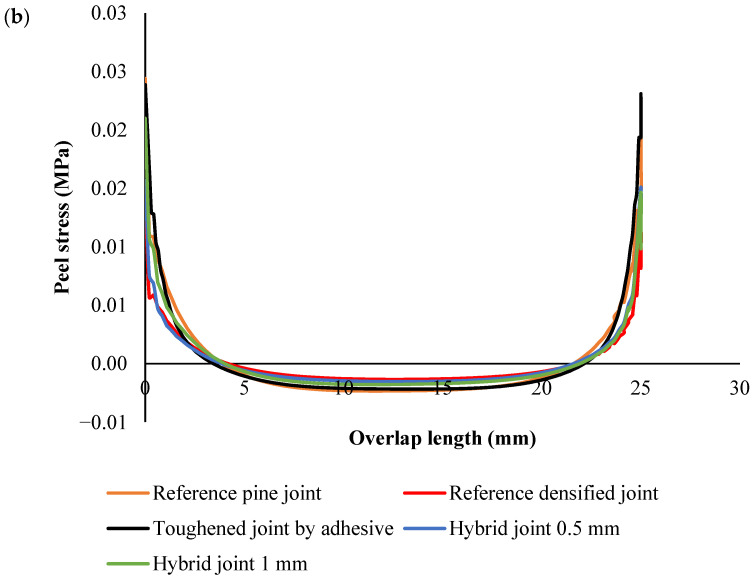
Peel stress distribution along the overlap length under static condition. Peel stress at the edges (**a**), and peel stress in total length (**b**).

**Figure 8:**
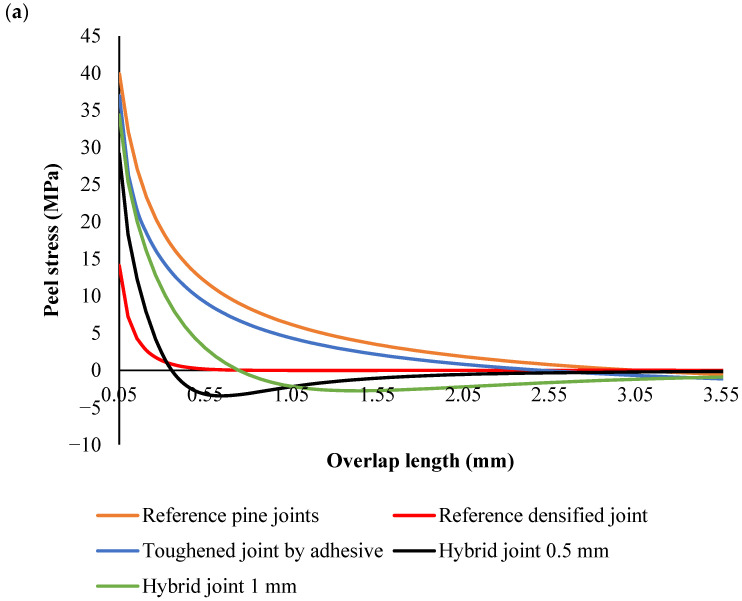

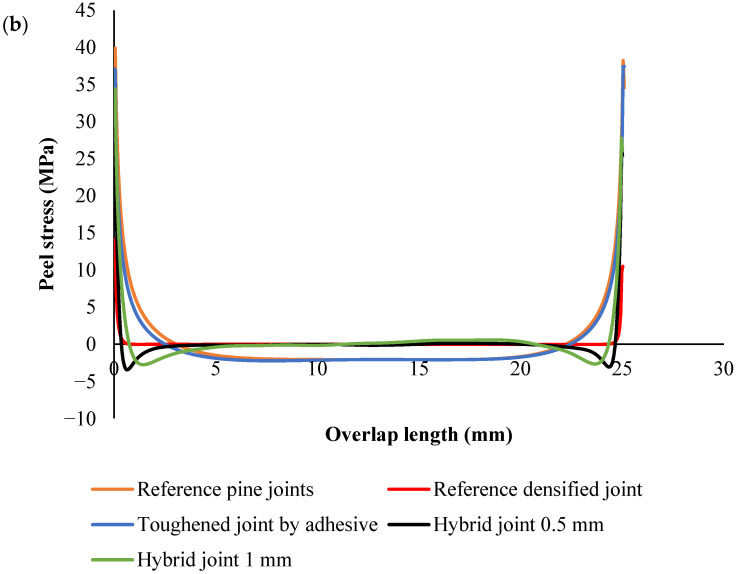
Peel stress distribution along the overlap length under intermediate rate conditions. Peel stress at the edges (**a**) and peel stress in total length (**b**).

Under static loading conditions, the trends were consistent with the dynamic case, with some differences in the peak stress values (Figure 7 and Figure 8). Reference pine exhibited the highest peak stress, indicating that under constant load, the pine wood substrate’s low stiffness led to greater stress concentrations near the overlap edges. This suggests that, under static conditions, untreated pine wood also has a higher risk of early failure at the overlap edges due to its greater susceptibility to deformation.

The toughened pine wood also showed reduced peak stress under static loading. The material’s increased toughness helped to distribute the load more evenly, resulting in lower peak stresses and a more even stress distribution along the overlap. Similarly, the densified pine wood exhibited the lowest peak stress, reflecting its higher stiffness and better ability to resist deformation under static loads, which led to a more uniform stress profile and reduced edge stress concentrations.

In the hybrid configurations, the static analysis showed similar behavior to the dynamic case. The hybrid joints with 0.5 mm thick toughened outer plies exhibited a relatively low peak stress, with a more even stress distribution. The hybrid joints with 1 mm thick toughened plies showed slightly higher peak stresses near the overlap edges compared to the 0.5 mm hybrid joints, but both configurations performed well in terms of distributing the load and reducing stress concentrations across the overlap. The hybrid design again demonstrated its ability to mitigate stress concentration and enhance joint performance, with a more balanced distribution of stress compared to the other configurations.

Peel stress analysis under dynamic loading conditions

**Figure 9:**
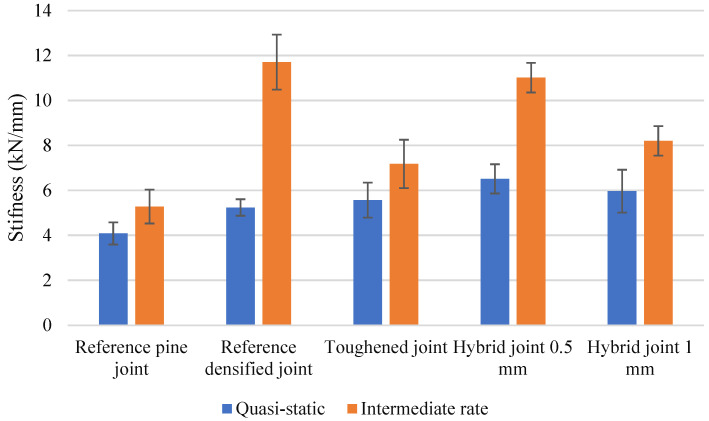
Stiffness variations under different loading conditions.

In the joints where reference pine wood was used as the substrate, peel stress exhibited the highest peak compared to all other configurations. The pine wood’s relatively low stiffness and flexibility led to substantial deformation under load. This deformation caused a sharp concentration of peel stress near the overlap edges, which is a typical behavior for materials with lower stiffness. The stress then dissipated gradually along the overlap as the pine wood deformed and spread the load. The high peak stress in this configuration indicates a greater susceptibility to failure at the overlap edges, where the stress concentration is most pronounced (Figure 9).

The toughening of pine wood was where the substrate was treated to absorb adhesive, making the wood tougher and more rigid. The toughened wood resulted in a reduction in peak peel stress compared to the reference pine wood. The adhesive absorption increased local stiffness, which helped mitigate the deformation near the overlap edges and led to a more even distribution of stress. While the peak stress was lower than in the reference joints, it was still relatively high compared to the other configurations, highlighting the improvement in material toughness but still exhibiting some stress concentration near the edges.

The densified pine joints had a much higher stiffness than both untreated and toughened pine wood, and as expected, the peak peel stress was the lowest among all the configurations. The high stiffness of the densified substrate resulted in less deformation under load, leading to a more even stress distribution (Figure 9). The densified wood resisted significant deformation, and while the stress concentrated near the overlap edges, it did so at a lower level than in the other configurations. This indicates that densified wood is more efficient at handling the applied load and reducing the likelihood of failure caused by stress concentration at the edges of the overlap.

In the hybrid design, a densified core and 0.5 mm thick toughened outer plies were used. The hybrid structure combined the high stiffness of the densified core with the flexibility and toughness of the toughened outer plies. The peak peel stress in this configuration was lower than in the densified wood alone, indicating that the addition of toughened outer plies helped distribute the stress more evenly across the overlap. A noticeable feature of this configuration was the negative peel stress near the overlap edges. This compressive stress near the edges is beneficial because it counteracts the tensile peel forces, which can cause adhesive failure or delamination. The tougher outer plies absorbed some of the stress, improving the overall load distribution and reducing the risk of failure at the overlap edges (Figure 9).

The hybrid joints, which used a densified core with 1 mm thick toughened outer plies, showed the lowest peak peel stress among all the configurations. The thicker outer plies helped redistribute the stress more effectively, leading to the most uniform stress profile across the overlap. The negative peel stress near the overlap edges was more pronounced in this configuration, further reducing the likelihood of adhesive failure and promoting a more durable adhesive bond. The thicker toughened plies in this configuration enhanced the stress redistribution even more, leading to a lower peak stress and a better overall performance in terms of both strength and durability.

Across all configurations, the stress distribution followed similar trends (Figure 8); the peak peel stress was concentrated near the edges of the overlap, and the stress in the middle of the joint was generally lower. In the densified and hybrid configurations, the stress at the middle of the overlap was close to zero MPa, indicating that the majority of the load was concentrated at the overlap edges, where stress concentrations are highest. This is typical for joints with stiff substrates, as they resist deformation and transfer the load more efficiently to the edges. In contrast, in the reference and toughened pine wood configurations, the stress at the middle of the joint was negative, reflecting the more significant deformation and flexibility of the substrate. This flexibility caused compressive forces to build up in the center of the overlap.

A key observation in the hybrid joints, particularly in Configurations 4 and 5, was the presence of negative peel stress near the edges of the overlap. This negative stress, which is indicative of compressive forces, plays a positive role in preventing adhesive failure. By counteracting the tensile peel stresses that typically cause delamination, the negative peel stress helps improve the bondline integrity and enhances the overall strength of the joint. The negative stress near the edges also promotes better load transfer across the overlap, helping to avoid premature failure.

The results also demonstrated that hybrid joints with a densified core and toughened outer plies provide the best performance in terms of stress distribution and adhesive bond durability. These hybrid configurations optimize load transfer by balancing stiffness and flexibility. The combination of the densified core’s stiffness and the toughened plies’ ability to absorb and redistribute stress leads to lower peak stresses, more uniform stress distribution, and a reduced risk of failure at the overlap edges. The negative peel stress at the edges of the hybrid joints further strengthens the bondline, making these joints more resistant to delamination and adhesive failure.

The use of an additional tough layer can significantly decrease stress concentration levels at the overlap ends, resulting in a more uniform stress distribution along the overlap. Additionally, the absorption of adhesive by the wood enhances the strength of the wood in the transverse directions. These factors contribute to the observed improvements in mechanical properties under various loading conditions.

### 4.1. Implications for Joint Design

From both the static and dynamic analyses, it is evident that substrate modifications play a crucial role in improving the performance of adhesive joints. Toughened joints enhance adhesion strength but also exhibit a higher risk of localized failure due to concentrated stresses, particularly at the overlap edges. Densified joints offer a more uniform stress distribution, reducing the likelihood of early failure and improving the overall durability of the joint. Hybrid joints, which combine the benefits of both toughened outer plies and a densified core, provide the best overall performance by balancing the advantages of both stiffness and toughness. The addition of toughened plies in the hybrid joints significantly mitigates stress concentrations, improving the load transfer across the bondline. The 1 mm thick outer ply offers enhanced stiffness and load distribution but may slightly increase the stress near the edges, suggesting that optimized hybrid designs can further improve wood-based adhesive joints.

Under dynamic conditions, the hybrid joints exhibit higher stress concentrations near the overlap edges and elevated overall stress levels compared to static conditions. This difference arises from the rapid application of the load (6 m/min displacement rate) and the strain rate sensitivity of the materials. At higher loading rates, the adhesive and substrate materials behave viscoelastically, temporarily stiffening and increasing their resistance to deformation. This stiffening effect amplifies stress levels, particularly in critical areas like the overlap edges, where load transfer is most concentrated. For hybrid joints, the stiffness mismatch between the densified core and toughened outer plies exacerbates stress concentrations under dynamic loading. The sharp transition in material properties at the interfaces further concentrates stress, amplifying its level near the edges. This combination of rapid loading, material stiffening, and stiffness mismatch explains the higher stress levels and more pronounced edge concentrations under dynamic conditions.

Under dynamic conditions, the rapid load application reduces the time available for stress to redistribute along the bondline. As a result, stress builds up quickly at localized points, leading to sharper stress concentrations at the overlap edges. In contrast, static loading involves a gradual application of force, allowing the adhesive and substrate to deform more elastically and providing sufficient time for stress redistribution. This results in a more uniform stress distribution and lower edge stress concentrations under static conditions.

### 4.2. Quasi-Static

**Figure 10:**
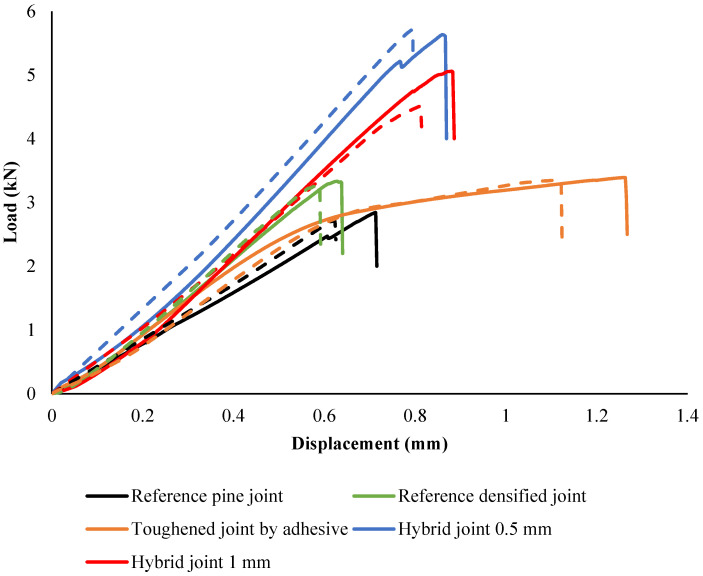
Load-displacement behavior of the joints under a static load. The dashed line represents the numerical behavior of the joints.

The experimental results shown in Figure 10 illustrate the load-displacement behavior of the joints under quasi-static conditions. It was found that the toughened joints had a failure load approximately 20% higher than the reference joints. Additionally, the joints exhibited a notable stiffening effect, with stiffness increasing by about 38%. Notably, at the point of failure, the toughened joints displayed plastic deformation within the joint structure when they reached the failure load of the reference joints. Consequently, the displacement at failure increased by around 85%, indicating a higher capacity for energy absorption before failure. This increase in absorbed energy was significant, approximately 170% more than the reference joints. The enhanced energy absorption capability of the toughened joints underscores their improved resilience and ability to dissipate energy effectively.

The nonlinear behavior of the joints results in a much larger area under the curve, leading to a substantial increase in the amount of energy absorbed during testing. Furthermore, it is important to note that the toughening process has a more significant impact on the absorbed energy of the joint than on its failure load, with the absorbed energy increasing by around 230%.

It was observed that the hybrid joints demonstrated the highest failure loads, with the hybrid joint (0.5 mm) reaching a peak load approximately 83% higher than the reference pine joint. The reference densified joint also showed a significant increase in strength, with a failure load approximately 50% higher than the pine joint.

The incorporation of hybrid joints with a toughened layer has been shown to effectively postpone failure, resulting in a substantial enhancement of joint strength by approximately 85% over standard reference joints. This significant increase can be attributed to the utilization of densified wood within the core layer, which bolsters the substrate’s strength, and the application of toughened wood combined with adhesive in the outer plies, which augments peeling strength. This dual approach, leveraging the strengths of both densified and toughened wood, achieves a synergistic effect that substantially elevates the overall structural integrity of the joints. The combination of these materials, each selected for their specific mechanical properties, has been meticulously engineered to work in concert, resulting in a composite material that not only meets but exceeds performance expectations in demanding applications. The displacement at failure exhibited notable differences among the joints. The joint toughened by adhesive showed a 37% increase in displacement compared to the reference pine joint, indicating enhanced flexibility. In comparison, the hybrid joints demonstrated similar or slightly greater displacement, with the 0.5 mm hybrid joint showing a displacement increase of approximately 38%, reflecting a greater capacity for energy absorption before failure. The failure modes of the joints were primarily brittle, with the exception of the reference densified joint, which exhibited a more gradual reduction in load post-peak, indicating a less brittle response. The stiffening effect observed in the densified joint reduced displacement by 25%, reflecting a trade-off between strength and flexibility. Of particular interest is the energy absorption capability of the hybrid joints. Due to the larger areas underneath their load–displacement curves, the hybrid joint (0.5 mm) absorbed approximately 83% more energy compared to the reference pine joint. This highlights the effectiveness of hybridization in enhancing both strength and energy dissipation capacity.

### 4.3. Fracture Surfaces

Analyzing fracture surfaces is essential for identifying failure causes in bonded joints. Fracture surfaces often exhibit micro and macro cracks. Examining crack initiation and propagation reveals key details about loading conditions and failure outcomes. In brittle materials, cracks typically initiate failure due to the absence of a plastic zone at the crack front, leading to rapid propagation and sudden failure.

**Figure 11:**
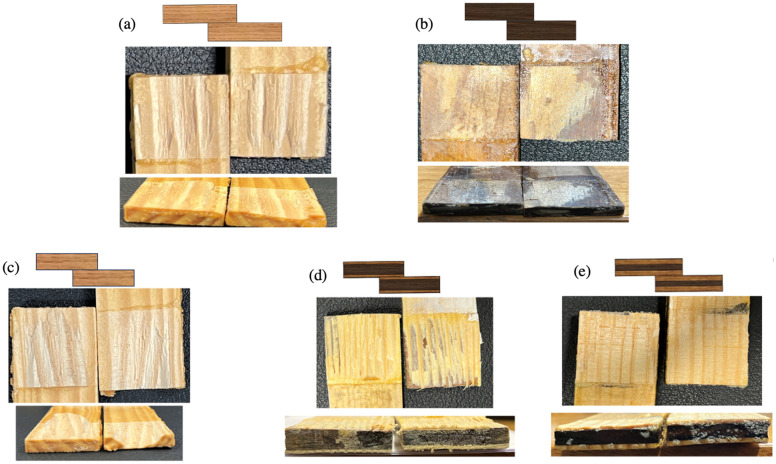
Joints’ fracture surfaces under static loads. Reference pine joint (**a**), reference densified joint (**b**), toughened joint (**c**), hybrid joint 0.5 mm (**d**), and hybrid joint 1 mm (**e**).

Figure 11 shows digital images of the fracture surfaces analyzed to understand joint behavior under quasi-static loads. The analysis revealed that wood delamination was the main failure mode in both toughened and reference joints, indicating effective adhesive curing and good adhesion. However, a significant difference was noted in the toughened joints compared with the reference joints. In the reference joints, delamination occurred at a shallow depth within the wood, concentrating failure in the surface layers. Conversely, the toughened joints exhibited a different failure pattern, with failure mainly occurring in the non-toughened wood region. This observation is significant because the non-toughened core bore most of the failure, while the outer layers, which had better adhesion due to adhesive penetration, were more resilient. This behavior increased the failure load of the toughened joints. The key to this phenomenon is the stress distribution along the overlap, where stress concentration at the ends approached the wood’s failure threshold, causing the observed change in failure mode. Figure 11 illustrates that in densified wood joints, substrate delamination was minimal due to the low peel strength of the densified wood. The substrate delaminated at the thickness with the highest stress level, close to the bonding line.

Figure 11 also highlights the different fracture behaviors of the joints. In the toughened joint, the crack path was more complex and deeper, requiring more energy to reach failure. In contrast, the reference joint had a relatively uniform delamination path, with the crack mainly propagating horizontally along the overlap. However, in the toughened joint, the crack followed a more intricate path, changing direction vertically and interacting with different grain orientations, even propagating vertically through the wood’s thickness. The delamination thickness in both hybrid joints was nearly identical; however, the joint with the 0.5 mm toughened layer displayed a more intricate fracture mechanism, which resulted in greater energy absorption during failure. This complexity can be attributed to the way the crack propagated through the material. Instead of following a straightforward path, the crack traveled between different wood grains and fibers, indicating a more irregular failure process. In some regions, the crack diverted or “kinked” toward the interface between the toughened layer and the densified wood, where the two materials were bonded together using the bio-adhesive.

This crack redirection suggests that the interaction between the toughened layer and the densified wood, facilitated by the bio-adhesive, played a significant role in resisting crack propagation. The presence of the toughened layer not only added complexity to the fracture process but also distributed the stresses across different planes, requiring more energy for the crack to progress. This intricate interaction between the wood fibers, toughened layer, and bio-adhesive likely contributed to the joint’s enhanced ability to absorb energy, making the fracture mechanism in the 0.5 mm hybrid joint more resistant to sudden failure compared to simpler joint configurations.

### 4.4. Intermediate Rate

#### 4.4.1. Load-Displacement Behavior

**Figure 12:**
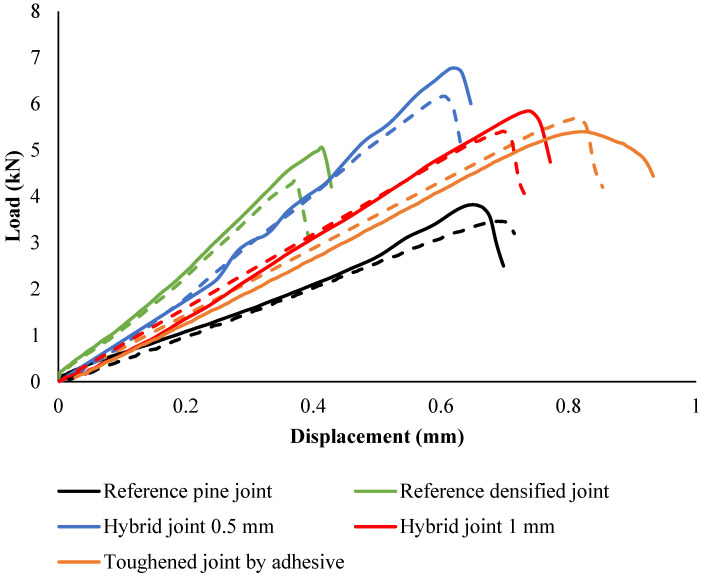
Load-displacement behavior of the joints under a high-rate load. The dashed line represents the numerical behavior of the joints.

Figure 12 illustrates the load-displacement behavior of the reference, toughened, densified, and hybrid joints under intermediate-rate conditions. Both experimental and numerical results reveal distinct differences in performance across configurations, highlighting the effectiveness of material modifications.

Experimental data indicate that toughened joints exhibit a 45% increase in failure load compared to reference joints, with a peak load of approximately 5.4 kN at 0.65 mm displacement. Numerical simulations corroborate this trend, accurately capturing the delayed damage initiation and higher energy absorption capabilities of toughened joints. These enhancements stem from the reinforcement provided by the toughening process, which improves the wood matrix’s resistance to delamination and adhesive failure. However, under intermediate-speed loading, both experimental and numerical results reveal a brittle failure mode characterized by abrupt post-peak stiffness degradation.

Densified joints perform moderately better than reference joints, achieving a peak load of 4.8 kN at 0.33 mm displacement. The densification increases stiffness and load-carrying capacity but does not enhance energy absorption to the same extent as toughened joints. Both experimental and numerical data confirm that densified joints exhibit a more gradual decline in stiffness after peak load, reducing the risk of sudden failure but lacking the toughness of toughened configurations.

Hybrid joints, which combine densified cores with toughened outer layers, offer a balance of stiffness and toughness. Experimental results show the 1 mm hybrid joint achieving a peak load of 5.7 kN at 0.6 mm displacement, while the 0.5 mm hybrid joint reaches 6.5 kN at 0.55 mm displacement. Numerical models align closely with these findings, reflecting the synergy between the core and outer layers that improves damage resistance and energy absorption. Hybrid joints exhibit more gradual stiffness degradation compared to fully toughened joints, making them a versatile choice for applications requiring a balance of load capacity and durability.

In contrast, reference joints display the lowest performance, with a peak load of approximately 3.7 kN at 0.45 mm displacement. Experimental and numerical results consistently reveal early damage initiation, primarily due to fiber tear-out, leading to rapid post-peak failure. This behavior underscores the limitations of untreated wood substrates under dynamic conditions.

#### 4.4.2. Damage Mechanisms and Failure Modes

**Figure 13:**
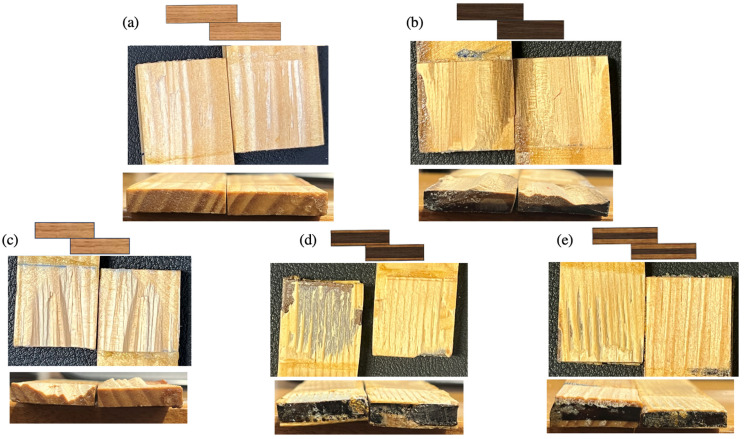
Fracture surface of SLJ under intermediate loading. Reference pine joint (**a**), reference densified joint (**b**), toughened joint (**c**), hybrid joint 0.5 mm (**d**), and hybrid joint 1 mm (**e**).

**Figure 14:**
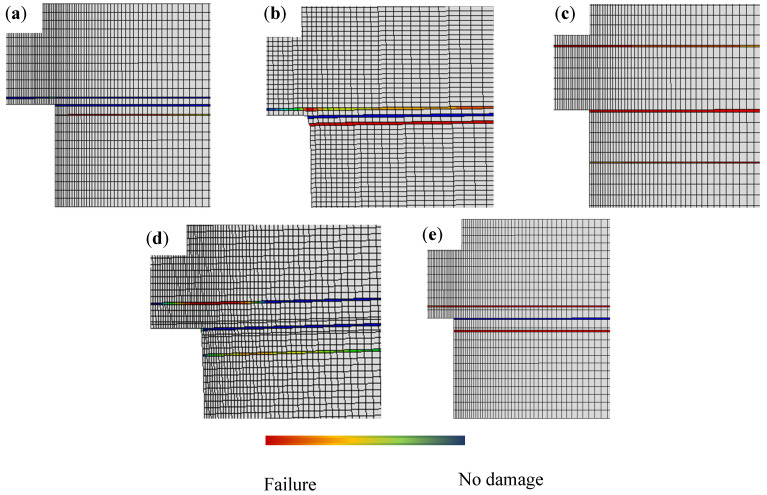
Damage parameter under intermediate loading. Reference joint (**a**), reference densified joint (**b**), toughened joint (**c**), hybrid joint 1 mm (**d**), and hybrid joint 0.5 mm (**e**).

The fracture surfaces, depicted in Figure 13, highlight the differences in damage mechanisms across joint configurations. Reference joints show minor delamination at the substrate interface, resulting in early failure. Conversely, toughened joints exhibit extensive delamination, attributed to improved peel strength and viscoelastic effects of lignin in the wood substrate. Numerical analysis supports these observations (Figure 14), showing delayed damage initiation in toughened joints, with cracks propagating through the substrate at higher loads.

Hybrid joints demonstrate a mix of delamination and fiber breakage. As loading rates increase, hybrid joints show deeper substrate delamination compared to reference and densified joints. The presence of toughened outer layers enhances peel strength, redirecting crack propagation through weaker transverse plies. This combination of behaviors, observed experimentally and predicted numerically, explains the hybrid joints’ improved performance under intermediate rates.

Densified joints show localized damage with less extensive delamination. Experimental and numerical analyses agree that densification reduces premature failure by creating a more uniform stress distribution across the bondline. However, this configuration lacks the toughness required to prevent failure at higher loads, as observed in the other configurations.

### 4.5. Strain Rate Sensitivity and Material Behavior

Under intermediate loading rates, viscoelastic materials like wood and bio-based adhesives exhibit increased stiffness but reduced ductility. Experimental data and numerical predictions consistently demonstrate that toughened joints withstand higher loads before damage initiation, with delayed microcracking and adhesive failure. Hybrid joints perform similarly, benefiting from the combined effects of toughened and densified components. Reference joints, on the other hand, show lower thresholds for damage initiation, with rapid crack propagation leading to early failure.

Delamination emerges as the dominant failure mode across all joint configurations. Toughened and hybrid joints resist delamination at higher loads, delaying the final failure. Reference joints, however, exhibit early delamination, resulting in a reduced load-carrying capacity. Numerical models accurately replicate these trends, validating the computational approach.

## 5. Conclusions

In this paper, the effects of a novel toughening method on the mechanical properties of wood joints bonded with bio-adhesive were investigated. The results showed that the toughening method significantly improved the failure load (60%), absorbed energy (130%), and stiffness (70%) of the joints under different loading conditions, such as quasi-static and intermediate-rate. The fracture surface analysis revealed that the toughening method changed the failure mechanism of the joints from delamination to fiber breakage, indicating that the strength of the substrate was lower than that of the joint under impact conditions. The viscoelastic behavior of the bio-adhesive also influenced the response of the joints to the changing displacement rate. The toughening method enhanced the resilience and load-bearing capacity of the wood joints, making them more suitable for dynamic applications. This paper contributes to the development of sustainable and durable wood joints using bio-based adhesives.

To further support these findings, SEM analysis of the phase interfaces in the adhesive joints was performed. The SEM analysis showed that the adhesive applied at the surface of the specimens penetrated the surface imperfections of the wood at the interface and filled the gaps. This process reduced the stress concentration and increased the energy dissipation at the crack tip, thereby enhancing the fracture toughness of the wood.

The combined experimental and numerical results highlight the superior performance of toughened and hybrid joints under intermediate loading rates. Toughened joints deliver the highest load-carrying capacity and energy absorption, albeit with more brittle failure modes. Hybrid joints, particularly the 1 mm configuration, offer a balanced response, combining the stiffness of densified cores with the toughness of toughened layers. Densified joints provide moderate improvements over reference joints but lack the enhanced toughness of the other configurations.

These findings emphasize the importance of tailoring joint designs to specific loading conditions. Toughening processes improve overall strength and durability, while hybridization offers an optimal combination of load capacity and resilience for dynamic applications. The close alignment between experimental and numerical results further reinforces the reliability of these insights for the design of advanced adhesive joints.

## Figures and Tables

**Table 1:** The elastic properties of pine wood were evaluated in three directions: longitudinal (L), radial (R), and tangential (T) [24].

*E*_L_ [GPa]	*E*_R_ [GPa]	*E*_T_ [GPa]	*ν*_LT_	*ν*_LR_	*ν*_TR_	*G*_LR_ [GPa]	*G*_LT_ [GPa]	*G*_TR_ [GPa]
12.0	1.9	1.0	0.5	0.4	0.3	1.1	1.0	0.3

**Table 2:** Strength (*σ*) properties of pine wood [24].

*σ*_L_ [MPa]	*σ*_R_ [MPa]	*σ*_T_ [MPa]	*σ*_LR_ [MPa]	*σ*_LT_ [MPa]	*σ*_RT_ [MPa]
97.5	7.9	4.2	16.0	16.0	4.5

**Table 3:** Bio-adhesive properties [25].

Young’s Modulus (*E*) [MPa]	Tensile Strength [MPa]	Mode I Fracture Energy (*G_IC_*) [N/mm]	Mode II Fracture Energy (*G_IIC_*) [N/mm]
197.09 ± 9.76	3.27 ± 0.14	0.33 ± 0.03	1.27 ± 0.1

**Table 4:** Elastic behavior of toughened wood and reference wood [26].

		Young’s Modulus [MPa]	Tensile Strength [MPa]	Strain at Failure [%]
Fiber direction	Reference	12.3 ± 1.2	93.2 ± 4.2	7.6 ± 0.3
	Toughened	11.9 ± 1.8	102.3 ± 8.2	1.3 ± 0.1
Matrix direction	Reference	2.1 ± 0.1	8.1 ± 0.3	0.4 ± 0.1
	Toughened	0.8 ± 0.1	11.8 ± 0.8	1.6 ± 0.1

## Data Availability

Data are contained within the article.
